# Supplementary material for: Abundance of Regulatory T Cell (Treg) as a Predictive Biomarker for Neoadjuvant Chemotherapy in Triple-Negative Breast Cancer
Source: Cancers (Basel). 2020 Oct 19;12(10):3038. doi: 10.3390/cancers12103038 (PMC7603157; doi:10.3390/cancers12103038)
Supplement: Supplementary file 1 [file cancers-12-03038-s001.pdf]

Abundance of Regulatory T Cell (Treg) as a Predictive Biomarker for Neoadjuvant Chemotherapy in Triple-Negative Breast Cancer

Masanori Oshi, Mariko Asaoka, Yoshihisa Tokumaru, Fernando A Angarita, Li Yan, Ryusei Matsuyama, Emese Zsiros, Takashi Ishikawa, Itaru Endo and Kazuaki Takabe

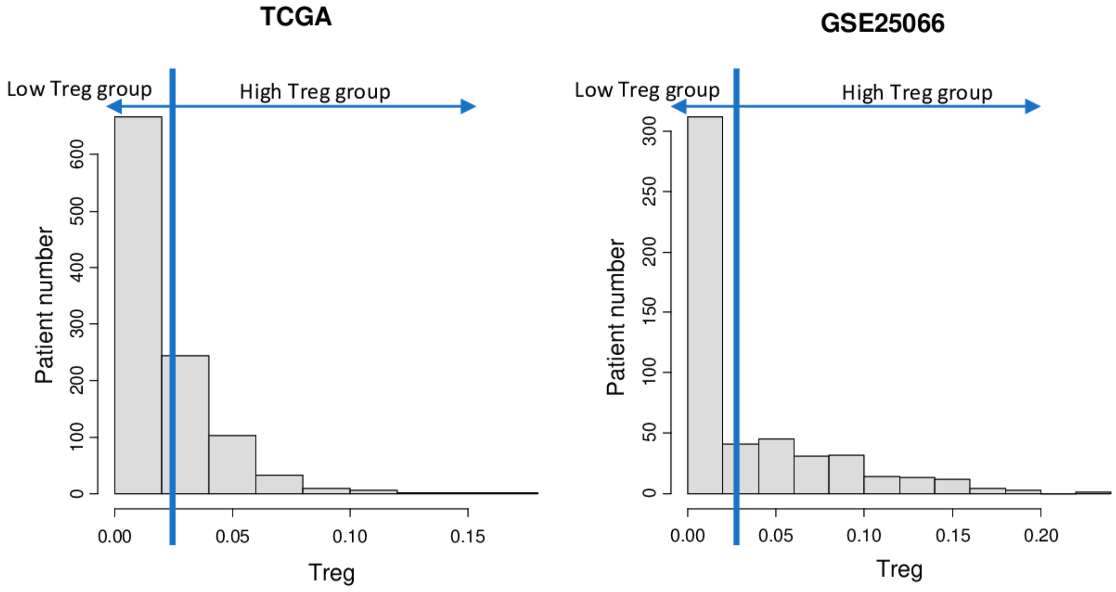

Figure S1. Histogram of Treg in TCGA and GSE25066 cohorts.

Table S1. Genes used to calculate the regulatory T cells (Tregs) score in the xCell method.

Gene Name	Gene Title
ATG2B	autophagy related 2B
BANP	BTG3 associated nuclear protein
CCR3	C-C motif chemokine receptor 3
CCR4	C-C motif chemokine receptor 4
CCR8	C-C motif chemokine receptor 8
CD28	CD28 molecule
CD5	CD5 molecule
CTLA4	cytotoxic T-lymphocyte associated protein 4
CXCR6	C-X-C motif chemokine receptor 6
FOXP3	forkhead box P3
GALNT8	polypeptide N-acetylgalactosaminyltransferase 8
GPR25	G protein-coupled receptor 25
HS3ST3B1	heparan sulfate-glucosamine 3-sulfotransferase 3B1
ICOS	inducible T cell costimulator
IKZF4	IKAROS family zinc finger 4
IL10RA	interleukin 10 receptor subunit alpha
IL2RA	interleukin 2 receptor subunit alpha
IPCEF1	interaction protein for cytohesin exchange factors 1
ITGB7	integrin subunit beta 7
KCNA2	potassium voltage-gated channel subfamily A member 2
LAIR2	Leukocyte Associated Immunoglobulin Like Receptor 2
LAX1	lymphocyte transmembrane adaptor 1
LRP2BP	LRP2 binding protein
MCF2L2	MCF.2 Cell Line Derived Transforming Sequence-Like 2
MCM9	minichromosome maintenance 9 homologous recombination repair factor
PLCL1	phospholipase C like 1
PPM1B	Protein phosphatase 1B
RGS1	regulator of G protein signaling 1
SIT1	signaling threshold regulating transmembrane adaptor 1
SPTAN1	spectrin alpha, non-erythrocytic 1
STAM	signal transducing adaptor molecule
TTN	tumor necrosis factor
TULP4	tubby like protein 4
UBE4A	ubiquitination factor E4A
VPS54	VPS54 subunit of GARP complex
ZCCHC8	zinc finger CCHC-type containing 8
ZFC3H1	zinc finger C3H1-type containing
ZMYM1	zinc finger MYM-type containing 1
ZNF236	zinc finger protein 236

Table S2. Genes used to calculate each immune cell, including CD8+ T cell, Cd4+ memory T cell, T helper type 1 and type 2 cell, M1 and M2 macrophages, dendritic cell (DC), and NK cell.

**CD8⁺ T cell**
AAK1, APBB1, ARHGEF1, BTN2A1, C7orf26, CA6, CASP8, CBY1, CCDC25, CCDC53, CCR7, CD160, CD27, CD3D, CD7, CD8A, CD8B, CD96, CEPT1, CIAPIN1, CLUAP1, COG2, COPZ1, CRTAM, CTSW, CX3CR1, DHX15, DIDO1, DNAJB1, DPP8, DSC1, EEF1D, EML3, FAM134C, FBXW4, FKTN, FNBP4, FTO, GGNBP2, GIMAP4, GJC2, GZMH, GZMK, GZMM, HNRNPA0, HNRNPL, IL16, IPCEF1, IRF3, KLHL3, KLRB1, KLRG1, KRT2, LAIR2, LSM14A, LY9, MED17, MKRN2, MMP19, MSL3, MTRF1, MYOM1, NAA16, NDFIP1, NDUFS2, NFKB1, NKRF, NPAT, NPRL2, PCNT, PFN2, PLCG1, PLXDC1, POLR3E, POP5, PRL, PRMT2, PRPF4B, PSD, PTGDR, PTPN4, PURA, RAPGEF6, RASA2, RBL2, RBM34, RING1, RNF113A, RPL37A, RWDD3, S100B, SDAD1, SDCCAG3, SFPQ, SHANK1, SIRPG, SLC1A7, SSTR3, TBCC, TMEM41B, TOMM7, TRAF3IP3, TSPAN32, TTN, UBE2Q1, UBQLN2, USP47, UTP20, WDR82, YLPM1, ZBTB11, ZC3HAV1, ZNF154, ZNF200, ZNF611, ZNF639
**CD4⁺ memory T cell**
AAMP, ACD, ACTL6A, ADSL, AHCTF1, AKT2, AMBRA1, ANP32B, ANXA7, API5, ARHGAP15, ARL2, ARPC4, ATF1, ATG5, ATPIF1, ATXN10, AURKAIP1, BAG3, BCAS2, BTF3, BUB3, C11orf58, C12orf29, CBLL1, CBX3, CCNC, CCR4, CD2, CD226, CD28, CD2AP, CD3G, CD40LG, CD5, CD6, CD96, CDC40, CDK9, CDKN2AIP, CDV3, CEP57, CETN3, CLPX, CMPK1, CNBP, COPS4, COPS5, COX7C, CPSF6, CSNK1A1, CSNK2A2, CTBP1, CTLA4, CXCR6, DAD1, DAP3, DBF4, DDX3X, DDX50, DENR, DLEC1, DNAJA2, DNAJB1, DOHH, DPM1, DR1, EEF2, EID1, EIF2B5, EIF3E, EIF3L, EIF3M, EIF4G2, ERH, ESD, ETAA1, EXOC2, FARS2, FCF1, FNTA, FUBP3, FXR1, GABPA, GALR2, GATAD2A, GLOD4, GLUD1, GPR132, GPR15, GPR171, GPR183, GRPEL1, GZMA, GZMK, HDAC1, HINT1, HMGB2, HMGN4, HMOX2, HNRNPA0, HNRNPH3, HNRNPU, ICOS, IMP3, INTS8, ISCA1, ITK, JAK3, KARS, KBTBD4, KIF22, KTN1, LDHB, LIMS1, LIN7C, MAEA, MAGOH, MATR3, MEN1, METAP1, METTL5, MMADHC, MRPL11, MRPL20, MRPL44, MRPS18B, MRPS27, MRPS34, NAE1, NCL, NDUFS5, NKRF, NUPL2, SGEP, PABPC4, PAPOLA, PCID2, PCNP, PDCD1, PDCD10, PFDN6, PFN1, PKP4, PLP2, POLD2, PPA2, PPID, PPIH, PPP1CB, PPP1CC, PPP2R5D, PPP6C, PREPL, PRPF18, PRPF19, PSMF1, PTGES3, PTPN11, PTPN4, RAD21, RANBP1, RANBP9, RBL2, RBM3, RBM34, RGS1, RNF34, RNF6, RPF1, RPL13, RPL13A, RPL36, RPL4, RPL5, RPL8, RPS19, RPS3, RPS6, RRP1B, RSL24D1, RUVBL1, RWDD1, SAFB2, SEC23IP, SERP1, SH2D1A, SLAMF1, SLC25A38, SLC25A6, SMAD2, SMC5, SMU1, SOD1, SP3, SPAG16, SRP9, SSNA1, STK16, SUB1, SUCLG1, SURF2, TBL3, THAP11, THOC7, THOP1, THRAP3, TINF2, TPP2, TPT1, TRA2A, TRAT1, TRMT112, TSN, TSSC1, TTC37, U2AF2, UBASH3A, UBE2D2, UBE2D3, UBE2N, UBIAD1, UBQLN2, UNC45A, UQCRC2, USP39, UXT, WDR46, ZC3H15, ZDHHC6, ZNF236, ZZZ3
**T helper type 1 cell**
TH1, CDC123, CHD1L, CHD4, COX10, CSTF1, CUEDC2, EIF2B2, FIBP, GNLY, HTRA2, IFNG, KIF20A, LAG3, MDC1, MNAT1, NCAPD3, NUP205, PKMYT1, POLD2, PPM1G, PSMD3, PTTG1, R3HDM1, RNPS1, RUVBL2, SLAMF1, SNRPC, TACO1, THOP1, TMEM39B, TRIM28, TTLL5, UBAP2, WDR18, WRAP53, ZBTB32
**T helper type 2 cell**
GZMK, IL5, IL13, MAD2L1, RRM2, BAG2, CXCR6, CEP55, RRAS2, NUP37, NPHP4, GPR15, GZMA, SMAD2, CDK2AP1, RGS9, SLC25A44, RAD50, TMEM39B, UBAP2, THADA, RNF34
**M1 macrophage**
ABCD1, ABI1, ABTB2, ACP2, ACTR2, ACTR3, ADAMDEC1, ADCK2, ADCY3, ADO, ADRA2B, AFG3L2, AGPS, ALCAM, ANXA2, AP1M2, ARHGEF11, ARL8B, ATOX1, ATP6V0C, ATP6V1A, ATP6V1D, ATP6V1E1, ATP6V1F, ATP6V1H, BCAP31, BCKDK, BLVRA, C1QA, C1QB, C3AR1, CALR, CCDC47, CCL1, CCL18, CCL19, CCL22, CCL24, CCL7, CCL8, CCR1, CD163, CD300C, CD48, CD63, CD80, CD84, CECR5, CHIT1, CIAO1, CLCN7, CLEC4E, CLPB, CLTC, CMKLR1, COQ2, CORO7, COX5B, CSF1, CSF1R, CXCL9, CYBB, CYC1, CYFIP1, CYP19A1, DAGLA, DLAT, DNAJC13, DNASE2B, DOT1L, EMILIN1, EXOC5, FAM32A, FANCE, FCER1G, FDX1, FKBP15, FOLR2, FPR2, FPR3, FTL, GLRX2, GP1BA, GPD1, HAMP, HAUS2, HEXB, HK3, HSPB7, HYAL2, IFNAR1, IGSF6, IL10, IL12B, IL17RA, ITGAE, ITGB1BP1, KCNJ1, KCNJ5, KCNK13, KIFC3, LAIR1, LAMP1, LILRB1, LILRB4, LIMD2, LONP1, LONRF3, MAPK13, MARCO, MFSD7, MMP19, MRPL12, MRPL40, MRS2, MS4A4A, MSR1, MT2A, MYBPH, MYH11, MYO7A, MYOF, MYOZ1, NARS, NCAPH, NDUFAF1, NDUFS2, NECAP2, NRBP1, OGFR, OTUD4, P2RX7, PDCL, PHLDB1, PKD2L1, PLEKHB2,

PQLC2, PRDX1, PTGIR, PTPRA, RAB3IL1, RELA, RNH1, RRP1, S100A11, S1PR2, SCAMP2, SDS, SIGLEC1, SIGLEC7, SIGLEC9, SLAMF8, SLC11A1, SLC1A2, SLC25A24, SLC31A1, SLC6A12, SNX3, SPG21, SPR, SRC, STIP1, STX12, STX4, TBC1D16, TCEB1, TDRD7, TFEC, TFRC, TIE1, TMEM33, TMEM70, TMX1, TPP1, TREM2, TRIP4, TSPO, UQCR11, USP14, UTP3, VIM, VPS33A, VSIG4, WDR11, WSB2, WTAP, ZC3H15, ZMPSTE24
**M2 macrophage**
ABCD1, ACP2, ACSM5, ADAMDEC1, ADCY3, ADRA2B, AGGF1, AKR7A2, ALDH9A1, ALG9, ALK, ANGPT4, ANKFY1, ANXA11, AP1B1, AQP8, ARFGEF2, ARHGEF11, ARSB, ATP2A2, ATP6V0A1, ATP6V0D1, ATP6V1C1, ATP6V1D, BAIAP2, BCAP31, BTBD1, C10orf76, C16orf62, CAMP, CANX, CARD14, CCDC85C, CCDC88A, CD52, CD63, CD81, CDS2, CEPT1, CLCN7, COL4A3BP, COMMD9, CYFIP1, DHX57, DNASE1L3, DNASE2B, EFR3A, ELK1, EXOC1, FDX1, FGR, FH, FKBP15, FLT1, FTL, GABARAP, GGA1, GLB1, GORASP1, GPD1, GSTO1, GUCA1A, HADHB, HAMP, HEXA, HEXB, HPS1, HS3ST2, HSPH1, IARS2, IFNAR1, IPPK, ITGAX, KCNJ1, KCNJ5, KCNK13, KCTD5, KIAA0196, LAIR1, LAMP1, LILRA2, LILRB4, LONRF3, MARCO, MFN1, MMP19, MRM1, MS4A4A, MSR1, MTMR14, MYO15A, MYO9B, MYOZ1, NAGPA, NCAPH, NCKAP1L, NDUFB1, NFS1, NOP10, NPR1, OS9, OSBPL11, P2RX7, PABPC4, PDCD6IP, PDE1B, PEX19, PICK1, PLEKHM2, POGK, PQLC2, RIN2, S100A6, SCAMP2, SDCBP, SDS, SLAMF8, SLC25A24, SLC25A46, SLC31A1, SLC38A7, SLC39A1, SLC6A12, SLC6A7, SLC9A6, SMG5, SNAPC2, SNX1, SNX2, SNX3, SNX5, SPG21, STX18, STX4, TAF10, TBC1D9B, TFEC, TMED5, TMEM184C, TMEM70, TMEM9B, TNFSF14, TPP1, TREM2, TSPO, UBXN6, UCP3, UGP2, UNC50, USF2, VPS35, VPS53, VSIG4, VTI1B, WDFY3, XPNPEP2, ZC3H3, ZCCHC4, ZNF219
**DC**
ACHE, ALCAM, ALDH1A2, ALOX15, ALOX15B, ARL8B, BCL2L11, BCL2L13, C1QA, C1QB, CAMK1G, CCDC81, CCL13, CCL17, CCL18, CCL19, CCL22, CCL23, CCL24, CCL8, CCR7, CD1A, CD1B, CD1C, CD1E, CD209, CD80, CD86, CD9, CEP350, CLEC10A, CUL1, DNASE1L3, DPYS, ETV3, F13A1, FBXL4, FCER2, FGL2, FPR3, GRIN1, GRSF1, GUCA1A, HCRTR2, HK3, HLA-DQA1, HPS5, HS3ST2, IL12B, IL21R, IRF4, KCNC3, KCNK13, KCNN1, LOR, MAP3K13, MAP3K6, MCF2, MPHOSPH6, MS4A4A, MS4A6A, NAGPA, NECAP2, NFKB1, NXPH3, PLD2, PRRG2, PTGES2, PTGIR, RAB8A, RNF2, RRP1B, SAMSN1, SIGLEC1, SLAMF1, SLAMF8, SLC30A4, SLCO5A1, SNX11, SPINT2, STAB1, SUZ12, TACSTD2, TBC1D13, TDRD7, TFEC, TMEM131, TMSB10, TNFRSF4, TRAF1, TREM2, TXN, UBE2Z, VAV2
**NK cell**
AGK, ALG13, AMZ2, ANKRD11, ARPC5L, ASTE1, BAD, BRD2, C1orf174, CCL4, CD160, CD244, CD247, CDKN2AIP, CHRNE, COQ10B, CTSW, CX3CR1, DNAJB14, DNAJC2, DR1, FASLG, FBXW4, FIP1L1, GGPS1, GIPR, GNA13, GNLY, GOLGA4, GPATCH8, GRIK4, GTF3C1, GZMB, GZMH, GZMM, HELZ, HIPK1, HIST1H3A, HNRNPL, IFNG, IL18RAP, IL21R, IL2RB, KLRD1, KLRG1, KPNB1, LAG3, LEMD3, LIM2, LTA, MAP3K7, MAPK1, MED1, MGAT2, MLH1, NCR1, NCR3, NEK1, NFE2L2, NKG7, NMUR1, OSBPL7, PJA2, PPP2CA, PRDM2, PRDX6, PRF1, PRKAG1, PTGDR, PTPN4, RAB14, RBM25, RBM39, RGS9, RSRC2, SACM1L, SBF1, SF3B4, SON, STAG2, STX8, SUPV3L1, TBCC, TBX21, THAP1, TKTL1, TNFSF11, TSPYL1, TSTD2, UBE2Q1, WBP11, WDR45, XCL1, YAF2, ZBTB1, ZBTB39, ZCCHC11, ZMYND11, ZNF264, ZNF426

Table S3. Available data of each cohort.

Cohort Name	Sample Number	NAC Response	Survival	Total Gene Number	CD274 Expression	Subtype	AJCC Stage	Grade	Metastatic Tumor	Mutation
TCGA	1065	−	OS, DFS, DSS	20501	+	+	+	*	−	+
GSE20194	248	+	−	13516	−	+	−	−	−	−
GSE25066	508	+	DFS	13516	−	+	+	+	−	−
GSE96058	3273	−	OS	30865	+	+	−	+	−	−
GSE110590	83	−	−	17096	+	−	−	−	+	−

* Manually identified by our group [1,2]; AJCC, American Joint Committee on Cancer; DFS, disease-free survival; DSS, disease-specific survival; NAC, neoadjuvant chemotherapy; OS, overall survival; TCGA, The Cancer Genome Atlas.

References

1. Asaoka, M.; Patnaik, S.K.; Zhang, F.; Ishikawa, T.; Takabe, K. Lymphovascular invasion in breast cancer is associated with gene expression signatures of cell proliferation but not lymphangiogenesis or immune response. *Breast Cancer Res. Treat.* **2020**, *181*, 309–322, doi:10.1007/s10549-020-05630-5.
2. Takahashi, H.; Oshi, M.; Asaoka, M.; Yan, L.; Endo, I.; Takabe, K. Molecular Biological Features of Nottingham Histological Grade 3 Breast Cancers. *Ann. Surg. Oncol.* **2020**, 10 1245 10434-, doi:10.1245/s10434-020-08608-1.

© 2020 by the authors. Licensee MDPI, Basel, Switzerland. This article is an open access article distributed under the terms and conditions of the Creative Commons Attribution (CC BY) license (http://creativecommons.org/licenses/by/4.0/).
